# Genomic and metabolomic insights into the enhancement of rare ginsenosides in *Panax ginseng* through solid-state fermentation by *Aspergillus cristatus* JH-5

**DOI:** 10.3389/fmicb.2026.1785529

**Published:** 2026-03-25

**Authors:** Fei Su, Tibin Duan, Rui Liu, Xulei Ding, Shuaishuai Mao, Yuqiang Zhang, Jinshuo Li, Qianzhen Wu, Chunjiang Zhang

**Affiliations:** 1 College of Life Sciences and Oceanography, Shenzhen University, Shenzhen, China; 2 Anhui Kanghe Traditional Chinese Medicine Technology Co. Ltd., Bozhou, Anhui, China; 3 School of Life Sciences, Lanzhou University, Lanzhou, China; 4 Gansu Key Laboratory of Biomonitoring and Bioremediation for Environmental Pollution, Lanzhou University, Lanzhou, China

**Keywords:** *Aspergillus cristatus*, ginsenoside Rg3, *Panax ginseng*, rare ginsenosides, solid-state fermentation

## Abstract

The therapeutic efficacy of *Panax ginseng* is often restricted by the low bioavailability of its naturally abundant major ginsenosides. Biotransformation into deglycosylated rare ginsenosides, such as Rg3, significantly enhances pharmacological activity. This study evaluates the potential of *Aspergillus cristatus* JH-5, a fungus with a long history of safe use in food fermentation isolated from Fuzhuan brick tea, as a biocatalyst for ginseng fermentation. We employed a multi-omics strategy integrating whole-genome sequencing (WGS), untargeted metabolomics, and HPLC quantification to elucidate the metabolic changes and underlying genetic mechanisms. Fermentation induced a profound restructuring of the ginseng metabolome. Most notably, HPLC analysis revealed the *de novo* accumulation of rare ginsenosides Rg3(S) and Rg3(R) to a total content of 0.23% (dry weight), compounds undetectable in the unfermented control. Genomic analysis of the 28.29 Mb JH-5 genome identified a rich arsenal of Carbohydrate-Active enZymes (CAZymes), specifically pinpointing multiple β-glucosidase candidate genes (e.g., gene0147, gene2409) responsible for the targeted deglycosylation of major saponins. Furthermore, the genome lacked biosynthetic gene clusters for common mycotoxins, supporting the strain’s safety profile for food applications. These findings establish *A. cristatus* JH-5 as a safe and efficient agent for enriching high-value rare ginsenosides, providing a solid theoretical and genetic basis for the development of novel fermented ginseng products.

## 1 Introduction

*Panax ginseng* C.A. Meyer has been revered for millennia in traditional East Asian medicine, where it is esteemed for its broad pharmacological effects, including immunomodulatory, anti-fatigue, and adaptogenic properties (Attele et al., 1999; Ratan et al., 2021). These pharmacological properties are primarily attributed to ginsenosides, a diverse class of triterpene saponins. While numerous ginsenosides have been identified, natural ginseng is dominated by “major ginsenosides,” such as Rb1, Rg1, and Re (Ru et al., 2015). However, the clinical efficacy of these naturally abundant compounds is often limited by their high molecular weight and bulky glycosyl moieties, which result in poor membrane permeability and low bioavailability in the human gastrointestinal tract (Kodli et al., 2025).

To address these pharmacokinetic limitations, pharmaceutical research has increasingly focused on “rare ginsenosides,” such as Rg3, Rh2, and Compound K. These deglycosylated derivatives are virtually absent in raw ginseng but exhibit significantly enhanced absorption rates and biological activities (Kim et al., 2015). Among these, Ginsenoside Rg3 and its dehydrated derivative Rg5 have garnered significant attention. Rg5, in particular, demonstrates superior therapeutic potential compared to its precursors, showing promise in inducing apoptosis in cancer cells and ameliorating neuroinflammation (Kim et al., 2017; Tanko et al., 2025; Chu et al., 2023). Consequently, the bioconversion of abundant major ginsenosides into high-value rare ginsenosides is a critical objective for the functional food industry.

Current conversion methods, such as physical steaming or chemical acid hydrolysis, often suffer from low specificity, environmental pollution, and the generation of undesirable by-products. In contrast, microbial biotransformation via solid-state fermentation (SSF) offers an eco-friendly and highly specific alternative (Zhang et al., 2024). Filamentous fungi are particularly advantageous in SSF due to their ability to penetrate solid substrates and secrete a diverse array of extracellular enzymes—such as β-glucosidases—which facilitate the targeted hydrolysis of glycosidic bonds (Verduzco-Oliva and Gutierrez-Uribe, 2020; Quan et al., 2012). This enzymatic approach allows for the precise structural modification of saponins while preserving their bioactivity.

*Aspergillus cristatus*, the dominant “Golden Flower” fungus involved in the fermentation of Fuzhuan brick tea, is a probiotic fungus with a proven safety profile (Rui et al., 2019). Genomic studies indicate that *A. cristatus* possesses rich biosynthetic gene clusters, including polyketide synthases (PKS) and glycosyl hydrolases, suggesting a robust potential for secondary metabolite modification (Ge et al., 2016; Guo et al., 2022). Despite its industrial importance in tea processing, the application of *A. cristatus* in the biotransformation of ginseng saponins remains underexplored, and the specific molecular mechanisms governing its metabolic interactions with terpene glycosides are not fully understood (Chen et al., 2023). Recently, the integration of multi-omics approaches—particularly genomics coupled with untargeted metabolomics—has emerged as a powerful tool to decode the complex biotransformation mechanisms of natural products during fermentation. Such integrative strategies have successfully elucidated novel metabolic pathways and identified key functional genes in various food matrices and functional ingredients (Shi et al., 2022; Ayon et al., 2024; Wei-Ye et al., 2024).

This study aims to establish an efficient bioprocess for enriching rare ginsenosides using a newly isolated strain, *Aspergillus cristatus* JH-5. We employed a comprehensive multi-omics framework, integrating whole-genome sequencing (WGS) to identify hydrolytic enzyme-coding genes and untargeted metabolomics to map metabolic flux during fermentation. Furthermore, High-Performance Liquid Chromatography (HPLC) was utilized to quantitatively evaluate bioconversion efficiency. Specifically, we monitored the degradation of major ginsenosides (Rg1, Rb1) and the targeted enhancement of rare ginsenosides (Rg3 and Rg5), providing theoretical insights for the industrial production of rare ginsenoside-enriched ginseng products.

## 2 Materials and methods

### 2.1 Experimental materials and reagents

Fuzhuan brick tea was purchased from the local tea market in Anhua, Hunan Province, China. *Panax ginseng* (dry root) was obtained from Jilin Tianbao Ginseng & Antler Co., Ltd. (Jilin, China) and authenticated as *Panax ginseng* C.A. Mey. SDAY medium was prepared with 40 g/L glucose, 10 g/L peptone, and 10 g/L yeast extract (adding 15 g/L agar for solid medium), and the pH was adjusted to 5.6 ± 0.2. Potato Dextrose Agar (PDA) and Potato Dextrose Broth (PDB) media were purchased from Guangdong Huankai Microbial Sci. & Tech. Co., Ltd. (China) and prepared according to the manufacturer’s instructions. Reference standards for ginsenosides Rg1, Re, Rd., Rg3(S), Rg5, and Rg3(R) (purity ≥ 98%) were obtained from Chengdu Ruifen Biotechnology Co., Ltd. HPLC-grade acetonitrile and methanol were used for chromatographic analysis, while other chemicals were of analytical grade.

### 2.2 Isolation, purification, and identification of *A. cristatus*

The isolation of the fungus was performed using a gradient dilution method. Briefly, 25 g of Fuzhuan brick tea was suspended in 225 mL of sterile 0.85% saline containing 12 g of glass beads (*Φ* 0.45 mm) and shaken at 28 °C and 180 rpm for 30 min. The supernatant was serially diluted (10^−1^ to 10^−6^), and 100 μL of each dilution was spread onto PDA and SDAY plates. The plates were incubated at 28 °C for 5–7 days. Distinct colonies were selected based on morphological characteristics and purified by repeated streaking on PDA plates. Among the multiple isolates obtained (e.g., JH-3, JH-4, JH-5, JH-9), strain JH-5 was specifically selected for subsequent fermentation studies due to its superior growth rate on ginseng powder as a sole carbon source in preliminary screening assays.

For morphological identification, purified strains were cultured on PDA at 28 °C, and colony characteristics (diameter, texture, color, and exudates) were recorded on days 4, 7, and 10. Microscopic features were examined by preparing wet mounts with sterile water. Hyphae, vesicles, cleistothecia, and ascospores were observed and photographed using an optical microscope.

Molecular identification was conducted by extracting genomic DNA from 7-day-old mycelia using a fungal DNA extraction kit (Guangzhou Lujia Biotechnology Co., Ltd.). The internal transcribed spacer (ITS) region was amplified using primers ITS1 and ITS4 (White et al., 1990). The PCR products were purified and sequenced by Tsingke Biotechnology Co., Ltd. The resulting sequences were compared against the NCBI database using BLASTn to determine taxonomic classification.

### 2.3 Complete genome sequencing and annotation

Strain JH-5 was cultured to the logarithmic growth phase, washed three times with phosphate-buffered saline (PBS), and fungal mycelia were collected by centrifugation at 12,000 rpm and 4 °C for 3 min. Genomic sequencing was performed by Shanghai Majorbio Bio-pharm Technology Co., Ltd. (China) on the Illumina HiSeq 4000 platform with 150 bp paired-end reads (PE150). A 400 bp paired-end library was constructed, and raw reads were assembled using SOAP.^1^ Assembly quality was assessed using BUSCO (using the fungi_odb10 dataset) and CEGMA. Functional annotation was performed using the KEGG, NR, Swiss-Prot, and Pfam databases. Orthologous groups were classified using eggNOG, and antibiotic resistance genes (ARGs) were identified using the Comprehensive Antibiotic Resistance Database (CARD).

### 2.4 Preparation of fermented ginseng

Solid-state fermentation was conducted using *A. cristatus* JH-5, based on the optimization strategies outlined by previous report (Xiao et al., 2022). Spore suspensions were prepared by adding sterile water to 6-day-old PDA cultures and gently scraping the surface. For substrate preparation, 20 g of ginseng powder was placed in a 250 mL Erlenmeyer flask and autoclaved at 115 °C for 30 min. The sterile ginseng was inoculated with the spore suspension of strain JH-5 at a final concentration of 1 × 10^6^ spores/g of substrate. A non-inoculated group injected with an equal volume of sterile water served as the control (CK). All flasks were sealed with sterile breathable sealing films to ensure aerobic conditions and incubated at 28 °C with 85% relative humidity for 14 days. During the fermentation period, the flasks were shaken manually under sterile conditions every 48 h to prevent mycelial clumping and ensure uniform oxygen distribution.

### 2.5 HPLC analysis of ginsenosides

Ginsenosides were extracted according to a modified method (Piao et al., 2020). Briefly, 1.0 g of sample powder was degreased with chloroform under reflux for 3 h. The residue was dried, extracted with 50 mL of water-saturated n-butanol overnight, and sonicated (250 W, 50 kHz) for 30 min. The filtrate was evaporated to dryness using a rotary evaporator, and the residue was dissolved in HPLC-grade methanol to a final volume of 5 mL. The solution was filtered through a 0.22 μm membrane prior to injection.

Quantification was performed using the external standard method. Calibration curves were constructed for Rg1, Rb1, Rg3(S), Rg3(R), and Rg5 with concentrations ranging from 0.05 to 1.0 mg/mL, all showing good linearity (R^2^ > 0.999). The Limits of Detection (LOD) and Quantification (LOQ) were determined at signal-to-noise (S/N) ratios of 3 and 10, respectively. The identification of rare ginsenosides (Rg3 stereoisomers) was confirmed by comparing retention times and UV spectra with authentic standards.

Analysis was performed on an Agilent 1260 HPLC system equipped with an Xtimate-C18 column (4.6 mm × 250 mm, 5 μm). The mobile phase consisted of acetonitrile (A) and 0.1% phosphoric acid in water (B). The gradient elution program was as follows: 0–20 min, 20% A; 20–30 min, 20%–35% A; 30–80 min, 35%–60% A; 80–90 min, 60% A; 90–100 min, 60%–90% A; 100–110 min, 90% A; 110–111 min, 90%–20% A; and 111–120 min, 20% A. The flow rate was 1.0 mL/min, the column temperature was maintained at 30 °C, the injection volume was 20 μL, and the detection wavelength was set at 203 nm.

### 2.6 Untargeted metabolomics analysis

Fermented samples were analyzed using a UHPLC-Q Exactive system (Thermo Fisher Scientific, USA). Chromatographic separation was achieved on a Waters HSS T3 column (100 mm × 2.1 mm, 1.8 μm). The mobile phases were 0.1% formic acid in water (A) and acetonitrile-isopropanol-water (47.5:47.5:5, v/v/v) containing 0.1% formic acid (B). Mass spectrometry data were acquired in full-scan mode (m/z 70–1,000) at a resolution of 70,000, followed by data-dependent MS/MS at a resolution of 17,500 with collision energies of 20, 40, and 60 eV. The ESI source parameters were: spray voltage 3,500 V (+)/2,800 V (−), sheath gas 40 psi, auxiliary gas 10 psi, and capillary temperature 400 °C. Data processing, including feature extraction and alignment, was performed using Agilent MassHunter Profinder (version 10.0), and metabolite annotation was conducted using Agilent Mass Profiler Professional (MPP) with the METLIN database.

For the qualitative analysis of LC–MS data, stringent criteria were applied to ensure reliability. A feature was considered reliably detected and its expression value retained for downstream analysis only if its signal-to-noise (S/N) ratio was ≥3; otherwise, the expression value was recorded as null. Furthermore, metabolite annotation against the database was performed using the following strict matching criteria: (1) a mass error of the precursor ion ≤10 ppm; (2) a mass error of the MS/MS spectra (fragment ions) ≤25 ppm; and (3) high isotopic pattern similarity.

### 2.7 Data availability

The raw sequencing data generated in this study have been deposited in the NCBI database and are publicly accessible at https://www.ncbi.nlm.nih.gov/. The ITS sequences for the *Aspergillus cristatus* strains have been assigned the following GenBank accession numbers: PX795264 (strain JH-5), PX795265 (strain JH-9), PX795266 (strain JH-3), and PX795267 (strain JH-4). The whole-genome sequence of *A. cristatus* JH-5 has been deposited under the accession number JBTJBX000000000.

### 2.8 Statistical analysis

All fermentation experiments, HPLC quantifications, and basic biochemical assays were performed with at least three independent biological replicates (*n* = 3). Data are presented as the mean ± standard deviation (SD). Statistical significance between the control and fermented groups was evaluated using the Student’s *t*-test in SPSS (version 26.0), with *p* < 0.05 considered statistically significant.

## 3 Results

### 3.1 Identification of strain JH-5

#### 3.1.1 Morphological identification

The colony morphology was observed on PDA medium after incubation at 25 °C for 7 days. The colonies were moderately deep with a slightly sulcate topography and entire margins. The mycelium appeared sulfur yellow to orange, exhibiting a velvety to floccose texture. The colony established a cream-yellow center with a light-yellow edge. Yellow pigments were observed without exudates, but brown exudates were secreted at the center of the colony if they continued to grow. The reverse side of the colony was straw to sulfur yellow or fulvous.

**Figure 1:**
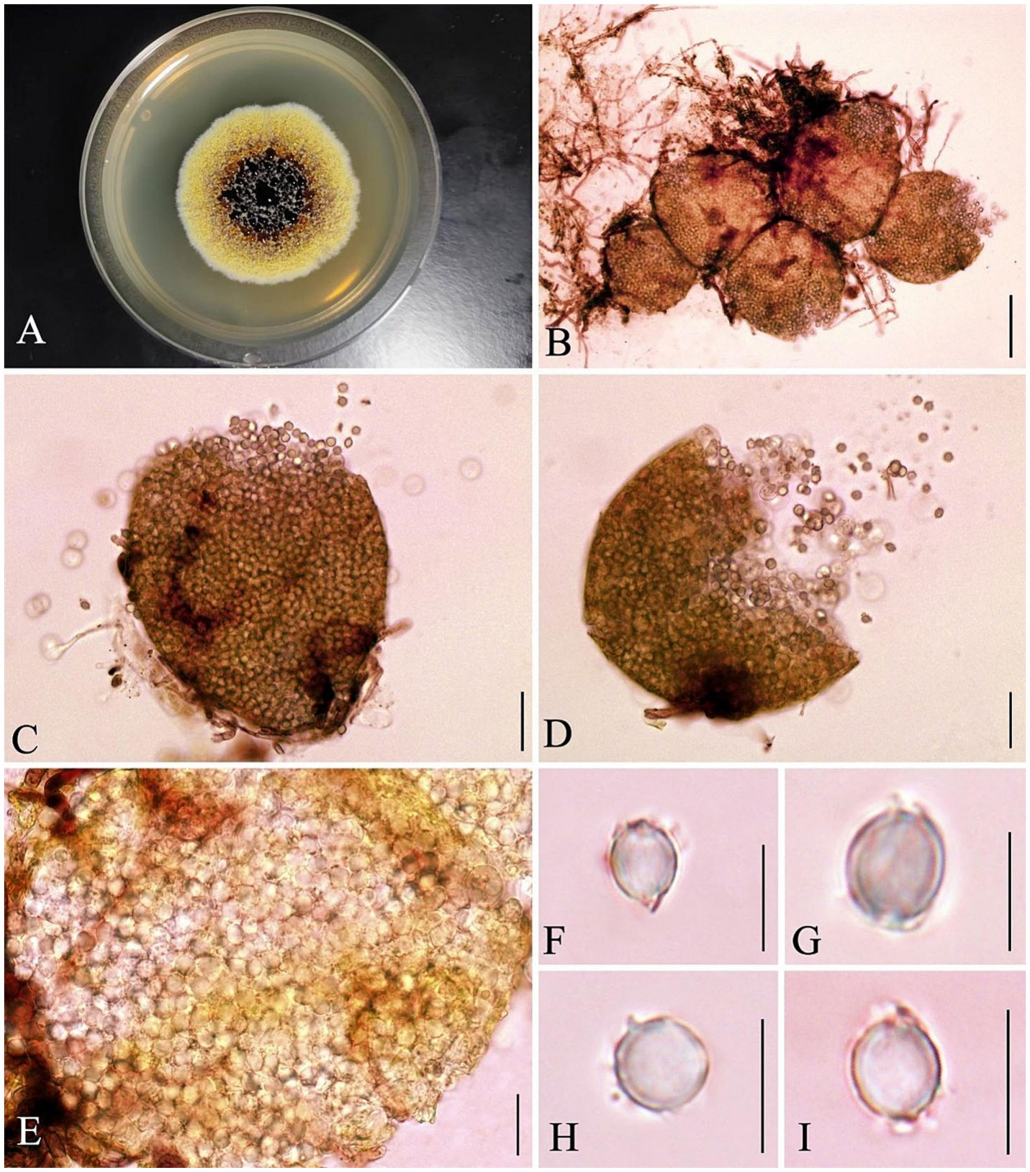
Morphological characteristics of *A. cristatus* JH-5. **(A)** Colony morphology on PDA after 7 days at 25 °C. **(B–D)** Ascomata. **(E–I)** Ascospores. Scale bars: **(B)** = 50 μm; **(C,D)** = 20 μm; **(E)** = 10 μm; **(F–I)** = 5 μm.

Micromorphologically, the strain produced eurotium-like, cleistothecial ascomata that were superficial, yellow, and globose to subglobose, with a diameter of 100–200 μm. The asci were 8-spored and globose to subglobose. Ascospores were hyaline; in surface view, they appeared globose to subglobose with verruculose to rugulose spore bodies (4.5–6 × 4.0–6 μm); in side view, they were lenticular with a distinct furrow and crests measuring 1.3–1.4 μm. Vesicles were globose to subglobose (36–50 μm wide) and fertile over two-thirds to the entire surface. Based on these macroscopic and microscopic characteristics, the strain was preliminarily identified as *Aspergillus cristatus* (see Figure 1).

#### 3.1.2 Molecular biology identification

**Figure 2:**
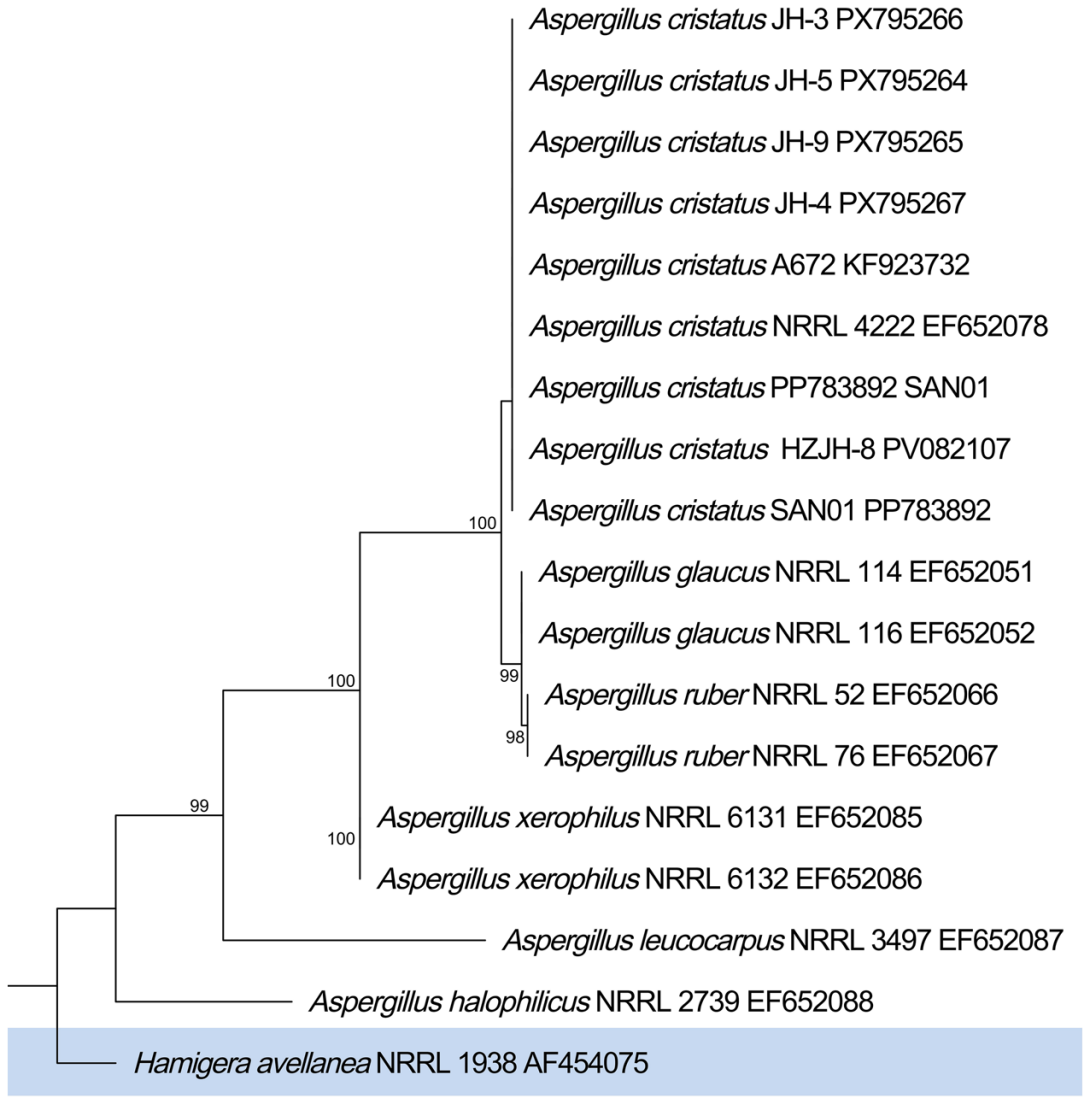
Phylogenetic tree based on ITS gene sequences, showing the evolutionary position of *Aspergillus cristatus* JH-5. The tree was constructed using the maximum likelihood method. Bootstrap values, based on 1,000 replicates, are shown at the nodes (ranging from 71 to 100). *Hamigera avellanea* NRRL 1938 was used as the outgroup to root the tree.

To corroborate the morphological identification, molecular classification was performed based on the internal transcribed spacer (ITS) region. The ITS sequences of four isolated strains were amplified, sequenced, and submitted to GenBank under the accession numbers PX795264 (strain JH-5), PX795265 (strain JH-9), PX795266 (strain JH-3), and PX795267 (strain JH-4). Phylogenetic analysis was conducted using MEGA X software. A phylogenetic tree was constructed using the Maximum Likelihood (ML) method to visualize the genetic relationships between the target strains and reference strains (Figure 2). The analysis revealed that the ITS sequence of strain JH-5 shared 100% similarity with *Aspergillus cristatus*. Consequently, based on the combined evidence from morphological observation and molecular phylogenetic analysis, the isolated strain JH-5 was definitively identified as *Aspergillus cristatus*.

### 3.2 Whole-genome sequencing and bioinformatics analysis of strain JH-5

#### 3.2.1 The basic information of whole genome

The whole genome of *A. cristatus* JH-5 was sequenced using NGS technology. A total of 4.76 Gb of raw data were generated. After quality filtering, 29.83 million clean pair reads were obtained (4.49 Gb), with Q20 and Q30 values of 99.44 and 96.79%, respectively. The final assembly consisted of 14 scaffolds with a total size of 28.29 Mb. The assembly quality was evidenced by an N50 length of 3.71 Mb, a largest scaffold of 5.59 Mb, and a 98.3% completeness score. The GC content was 49.76%, and the gap rate (N rate) was 0%. Additionally, repetitive sequences constituted 2.74 Mb (9.67% of the genome), primarily comprising 5,935 simple repeats, 1,262 LTRs, 910 LINEs, and 870 DNA transposons. Detailed assembly statistics are listed in Table 1.

**Table 1:** Genome assembly details for strain JH-5.

Scaffold ID	Sample name	Length (bp)	G + C (%)	Bases statistics
Scaffold1	JH_5	5,587,160	49.82	A:1396967; T:1406772; G:1394533; C:1388888
Scaffold2	JH_5	5,097,015	49.93	A:1275532; T:1276773; G:1275386; C:1269324
Scaffold3	JH_5	3,711,547	49.82	A:931032; T:931309; G:922084; C:927122
Scaffold4	JH_5	3,699,369	49.81	A:926419; T:930146; G:920015; C:922789
Scaffold5	JH_5	2,656,406	49.46	A:669002; T:673545; G:655179; C:658680
Scaffold6	JH_5	2,619,893	49.87	A:655986; T:657260; G:651692; C:654955
Scaffold7	JH_5	2,354,658	49.9	A:586281; T:593440; G:586320; C:588617
Scaffold8	JH_5	2,006,522	49.5	A:505383; T:507958; G:495749; C:497432
Scaffold9	JH_5	292,979	50.53	A:71854; T:73077; G:73109; C:74939
Scaffold10	JH_5	120,841	50.59	A:30472; T:29231; G:31161; C:29977
Scaffold11	JH_5	79,401	28.28	A:27059; T:29886; G:10046; C:12410
Scaffold12	JH_5	26,129	57.35	A:5657; T:5487; G:8281; C:6704
Scaffold13	JH_5	17,363	57.18	A:3692; T:3742; G:4444; C:5485
Scaffold14	JH_5	16,152	57.82	A:3454; T:3359; G:5159; C:4180

#### 3.2.2 Genome functional annotation

Functional annotation was performed by aligning predicted gene sequences against public databases including NR, Swiss-Prot, Pfam, COG, GO, and KEGG. A total of 9,658 genes were successfully annotated. The detailed statistics are presented in Table 2. The NR database provided the highest annotation coverage (9,257 genes, 95.85%), followed by GO (7,007 genes, 72.56%) and Pfam (6,882 genes, 71.27%). Furthermore, SignalP analysis identified 648 genes containing signal peptides, representing 6.71% of the total coding sequences.

**Table 2:** Distribution of gene functional annotation databases for strain JH-5.

Type	Gene number	Annotation ratio (%)
Total gene number	9,658	100
NR	9,257	95.85
GO	7,007	72.56
Pfam	6,882	71.27
Swiss-Prot	6,616	68.50
KEGG	6,173	63.93
COG	3,372	34.91
PHI	2,361	24.45
TCDB	1,400	14.50
DFVF	1,010	10.46
Tmhmm	920	9.52
SignalP	648	6.71
CARD	152	1.57

#### 3.2.3 GO functional annotation

**Figure 3:**
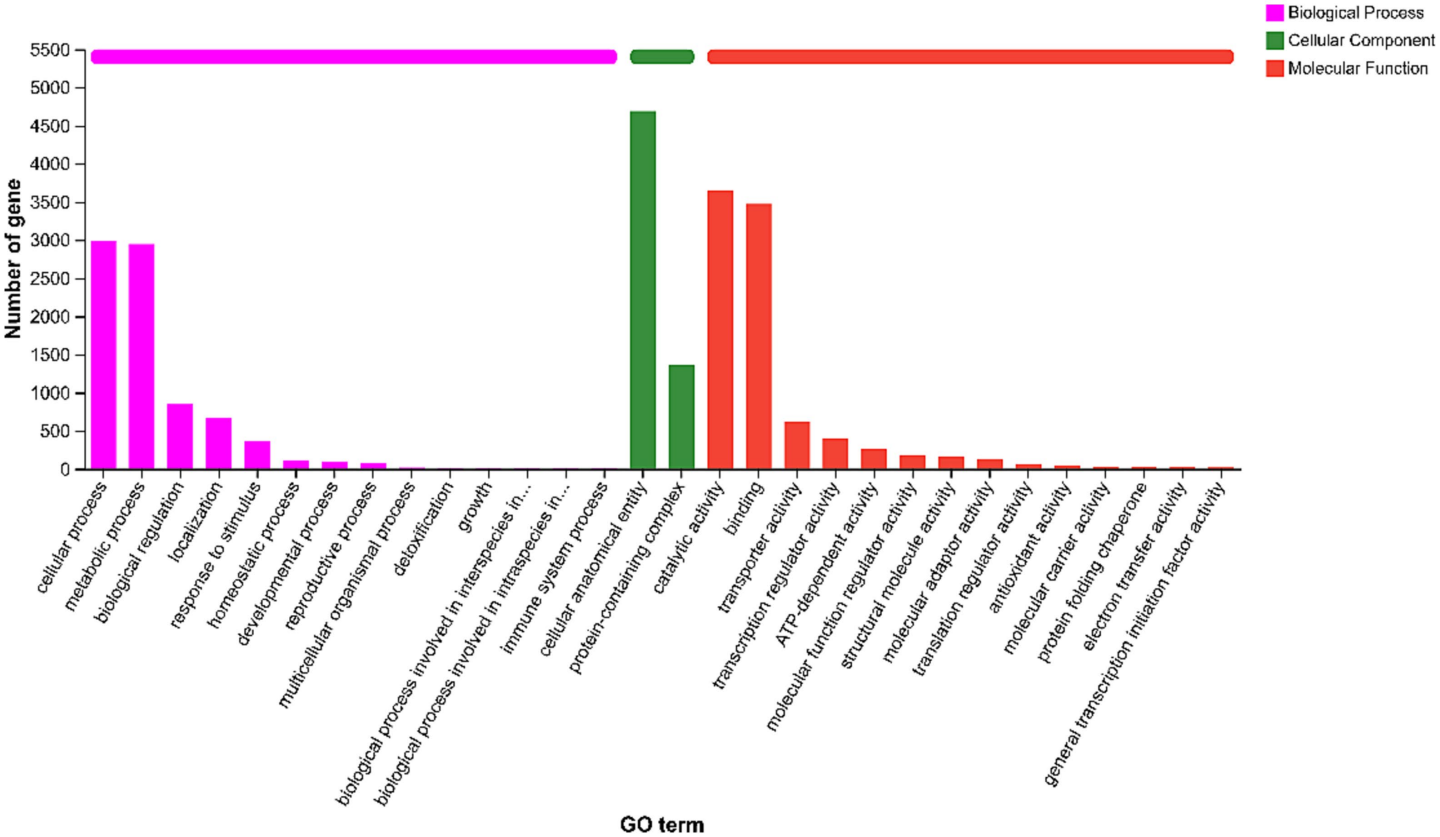
Gene ontology (GO) functional classification of the *Aspergillus cristatus* JH-5 genome. The *x*-axis indicates the GO terms (Level 2), and the *y*-axis represents the number of genes annotated to each term. The bar colors represent the three main ontological categories: purple for Biological Process (BP), green for Cellular Component (CC), and red for Molecular Function (MF).

Gene Ontology (GO) analysis classified 7,007 genes into three main categories: Biological Process (BP), Cellular Component (CC), and Molecular Function (MF) (Figure 3).

In the BP category, the most abundant terms were cellular process (2,994 genes) and metabolic process (2,945 genes), followed by biological regulation (848 genes) and response to stimulus (367 genes).

In the MF category, genes were predominantly enriched in catalytic activity (3,654 genes) and binding (3,471 genes). Notably, 613 genes were associated with transporter activity, and 38 genes were identified with antioxidant activity.

In the CC category, the majority of genes were assigned to cellular anatomical entity (4,688 genes) and protein-containing complex (1,361 genes). Detailed GO classification counts are listed in Table 3.

**Table 3:** GO functional classification of the *Aspergillus cristatus* JH-5 genome.

Category	GO term (level 2)	GO ID	Gene number
Biological process	Cellular process	GO:0009987	2,994
	Metabolic process	GO:0008152	2,945
	Biological regulation	GO:0065007	848
	Localization	GO:0051179	668
	Response to stimulus	GO:0050896	367
	Homeostatic process	GO:0042592	115
	Developmental process	GO:0032502	100
	Reproductive process	GO:0022414	80
	Multicellular organismal process	GO:0032501	16
	Detoxification	GO:0098754	12
	Growth	GO:0040007	9
	Interspecies interaction between organisms	GO:0044419	8
	Intraspecies interaction between organisms	GO:0051703	3
	Rhythmic process	GO:0048511	2
	Immune system process	GO:0002376	2
Cellular component	Cellular anatomical entity	GO:0110165	4,688
	Protein-containing complex	GO:0032991	1,361
Molecular function	Catalytic activity	GO:0003824	3,654
	Binding	GO:0005488	3,471
	Transporter activity	GO:0005215	613
	Transcription regulator activity	GO:0140110	392
	ATP-dependent activity	GO:0140657	266
	Molecular function regulator activity	GO:0098772	182
	Structural molecule activity	GO:0005198	157
	Molecular adaptor activity	GO:0060090	121
	Translation regulator activity	GO:0045182	66
	Antioxidant activity	GO:0016209	38
	Molecular carrier activity	GO:0140104	23
	Electron transfer activity	GO:0009055	20
	General transcription initiation factor activity	GO:0140223	18
	Cytoskeletal motor activity	GO:0003774	17
	Molecular transducer activity	GO:0060089	17
	Protein folding chaperone	GO:0044183	22

#### 3.2.4 COG functional annotation

**Figure 4:**
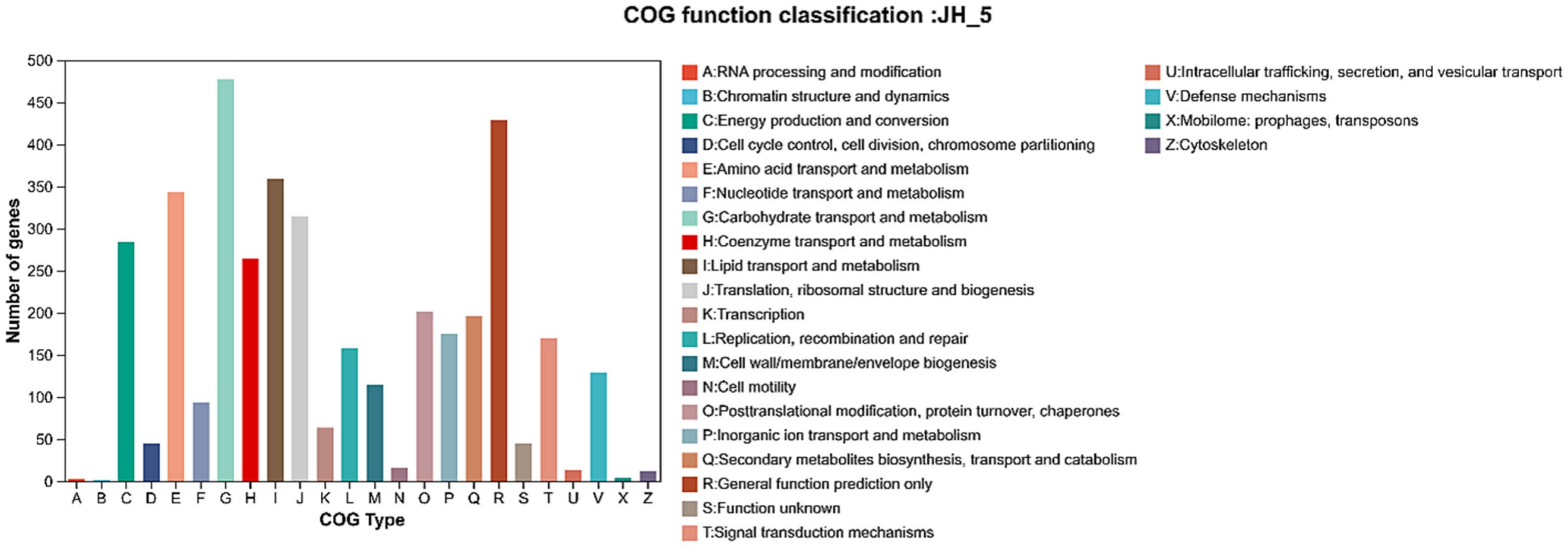
COG functional classification of the *Aspergillus cristatus* JH-5 genome. The horizontal axis represents the number of genes, and the vertical axis represents the functional categories (A–Z).

The Cluster of Orthologous Groups (COG) database was used to classify 3,372 protein-coding genes into 24 functional categories (Figure 4).

The “Metabolism” cluster represented the largest functional group. Within this cluster, Carbohydrate transport and metabolism (Category G) was highly enriched. Specific analysis of this category identified genes encoding glycoside hydrolases, including putative beta-glucosidases (e.g., gene0147, gene2409, gene7291) and aryl-phospho-beta-D-glucosidases (e.g., gene0541, gene2360).

Other significantly enriched categories included Secondary metabolites biosynthesis, transport, and catabolism (Category Q) and Lipid transport and metabolism (Category I). These categories contained genes encoding Cytochrome P450s (e.g., gene0175, gene3093) and UDP-glycosyltransferases (e.g., gene0843). Additionally, transporters classified under Inorganic ion transport and metabolism (Category P), such as MFS-type transporters (e.g., gene0109), were also identified.

#### 3.2.5 KEGG functional annotation

**Figure 5:**
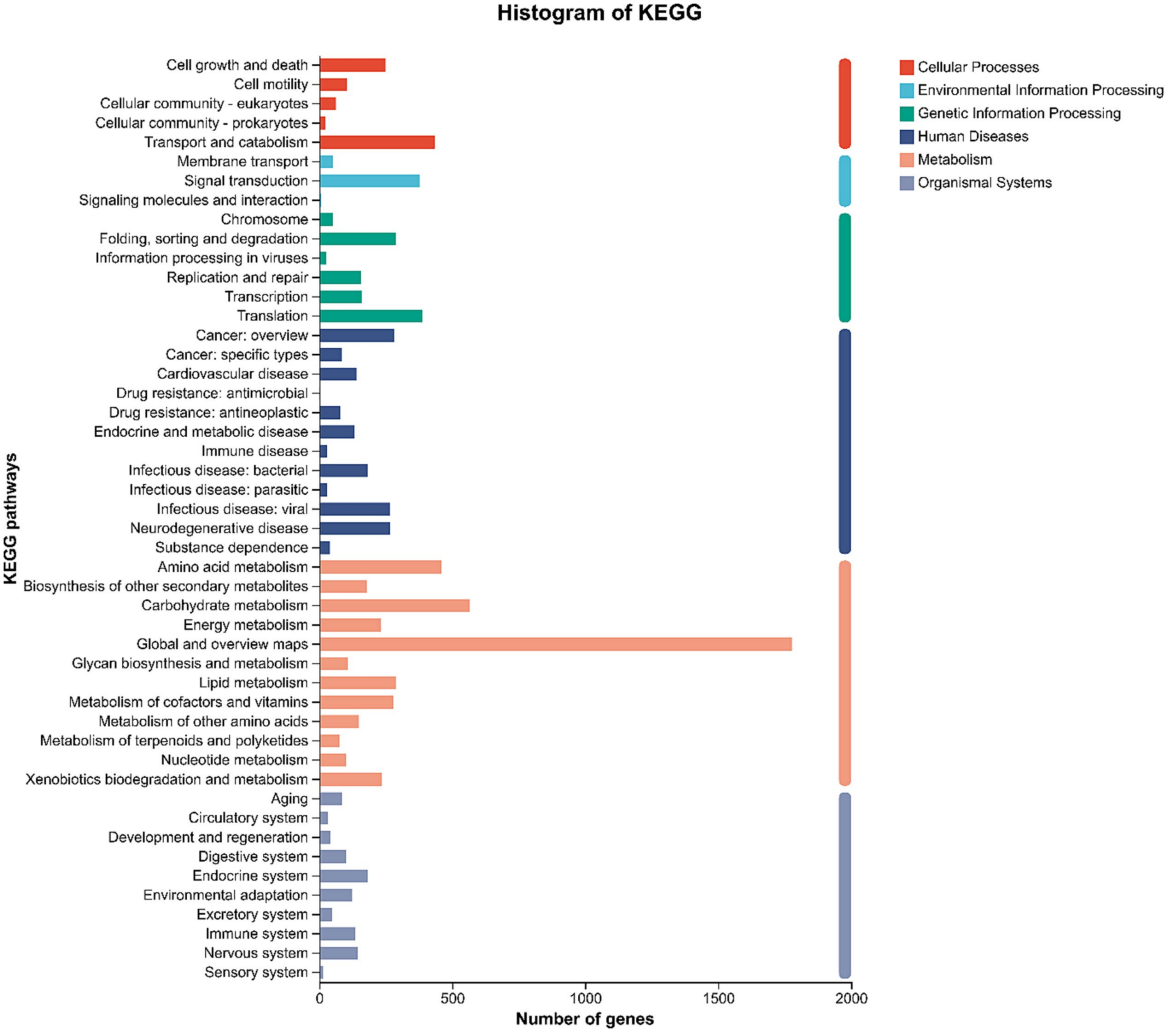
KEGG pathway classification of the *Aspergillus cristatus* JH-5 genome. The *y*-axis represents the KEGG functional categories (Level 2), and the *x*-axis represents the number of genes annotated to each category. The pathways are grouped by Level 1 categories (Metabolism, Genetic Information Processing, Environmental Information Processing, Cellular Processes, Organismal Systems, and Human Diseases).

KEGG pathway analysis mapped the annotated genes to five major categories (Figure 5). “Metabolism” was the dominant cluster, with Global and overview maps (1,777 genes), Carbohydrate metabolism (566 genes), and Amino acid metabolism (460 genes) being the top enriched sub-categories. Furthermore, 236 genes were mapped to Xenobiotics biodegradation and metabolism, and 75 genes to Metabolism of terpenoids and polyketides.

Key candidate genes potentially involved in ginsenoside bioconversion were identified (Table 4). These included multiple beta-glucosidases (EC:3.2.1.21; e.g., gene0147, gene2409) and glucan 1,3-beta-glucosidases (e.g., gene0541). Additionally, the genome encodes various Cytochrome P450s (e.g., gene0313) and MFS-type transporters (e.g., gene0109) (Ge et al., 2016).

**Table 4:** Key candidate genes in *A. cristatus* JH-5 involved in ginsenoside bioconversion identified via KEGG.

Gene ID	Gene name	KO ID	Enzyme/Protein description	Proposed function
*gene0147*	*bglX*	K05349	Beta-glucosidase [EC:3.2.1.21]	Key Enzyme: Hydrolyzing outer glucose of Rb1/Rd. to form F2/C-K
*gene2409*	*bglX*	K05349	Beta-glucosidase [EC:3.2.1.21]	Key Enzyme: Hydrolyzing outer glucose of Rb1/Rd. to form F2/C-K.
*gene7291*	*bglX*	K05349	Beta-glucosidase [EC:3.2.1.21]	Key Enzyme: Hydrolyzing outer glucose of Rb1/Rd. to form F2/C-K.
*gene0541*	*E3.2.1.58*	K01210	Glucan 1,3-beta-glucosidase	Hydrolysis of specific glucan linkages.
*gene0327*	*CLOA*	K25463	Cytochrome P450 monooxygenase	Potential modification of saponin aglycone backbone.
*gene0313*	*CYP619*	K21293	Cytochrome P450 family 619	Potential modification of saponin aglycone backbone.
*gene0109*	*GLIA*	K22134	MFS transporter, DHA2 family	Efflux/Transport of fermentation products.
*gene0012*	*DAL5*	K08192	MFS transporter, ACS family	Transmembrane transport of substrates.

#### 3.2.6 NR and Swiss-Prot database annotation

Comparison against the NR database annotated 8,456 genes (91.6% coverage). As shown in Table 5, the top functional category was Carbohydrate transport and metabolism (1,234 genes, 14.6%), followed by Amino acid transport and metabolism (892 genes, 10.6%).

**Table 5:** Top 20 distribution of NR database functional annotations for strain JH-5 genome.

Rank	Functional category	Number of genes	Percentage (%)
1	Carbohydrate transport and metabolism	1,234	14.6
2	Amino acid transport and metabolism	892	10.6
3	Translation, ribosomal structure	756	8.9
4	Energy production and conversion	678	8.0
5	General function prediction only	623	7.4
6	Transcription	512	6.1
7	Replication, recombination, repair	456	5.4
8	Lipid transport and metabolism	389	4.6
9	Cell wall/membrane/envelope biogenesis	345	4.1
10	Coenzyme transport and metabolism	301	3.6
11	Inorganic ion transport and metabolism	278	3.3
12	Secondary metabolites biosynthesis	256	3.0
13	Signal transduction mechanisms	234	2.8
14	Nucleotide transport and metabolism	212	2.5
15	Posttranslational modification	189	2.2
16	Intracellular trafficking	167	2.0
17	RNA processing and modification	145	1.7
18	Cell cycle control and mitosis	123	1.5
19	Defense mechanisms	98	1.2
20	Chromatin structure and dynamics	76	0.9

Annotation against the Swiss-Prot database covered 7,892 genes (85.5%). This analysis identified 128 genes encoding glycosyl hydrolases, including GH3 family β-glucosidases, GH5 cellulases, and GH2 β-galactosidases. Additionally, 45 genes were annotated to terpenoid backbone biosynthesis (ko00900).

#### 3.2.7 Secretome, transporter, and virulence factor analysis

The genome analysis predicted 809 secreted proteins, accounting for 8.38% of the total proteome, which includes various CAZymes and proteases (e.g., Sed1). Transmembrane prediction identified 2,246 proteins with transmembrane helices, and 768 genes showed homology to transporters in the TCDB.

Regarding safety assessment, 468 genes showed homologs in the Virulence Factor Database (VFDB). These genes primarily encoded conserved housekeeping proteins such as transcription factors (e.g., Tup1) and metabolic enzymes (e.g., Leu2), and no gene clusters associated with mycotoxins (e.g., aflatoxins or ochratoxins) were detected. The Comprehensive Antibiotic Resistance Database (CARD) analysis identified 290 genes with homology to antibiotic resistance markers.

### 3.3 Metabolomic analysis

**Figure 6:**
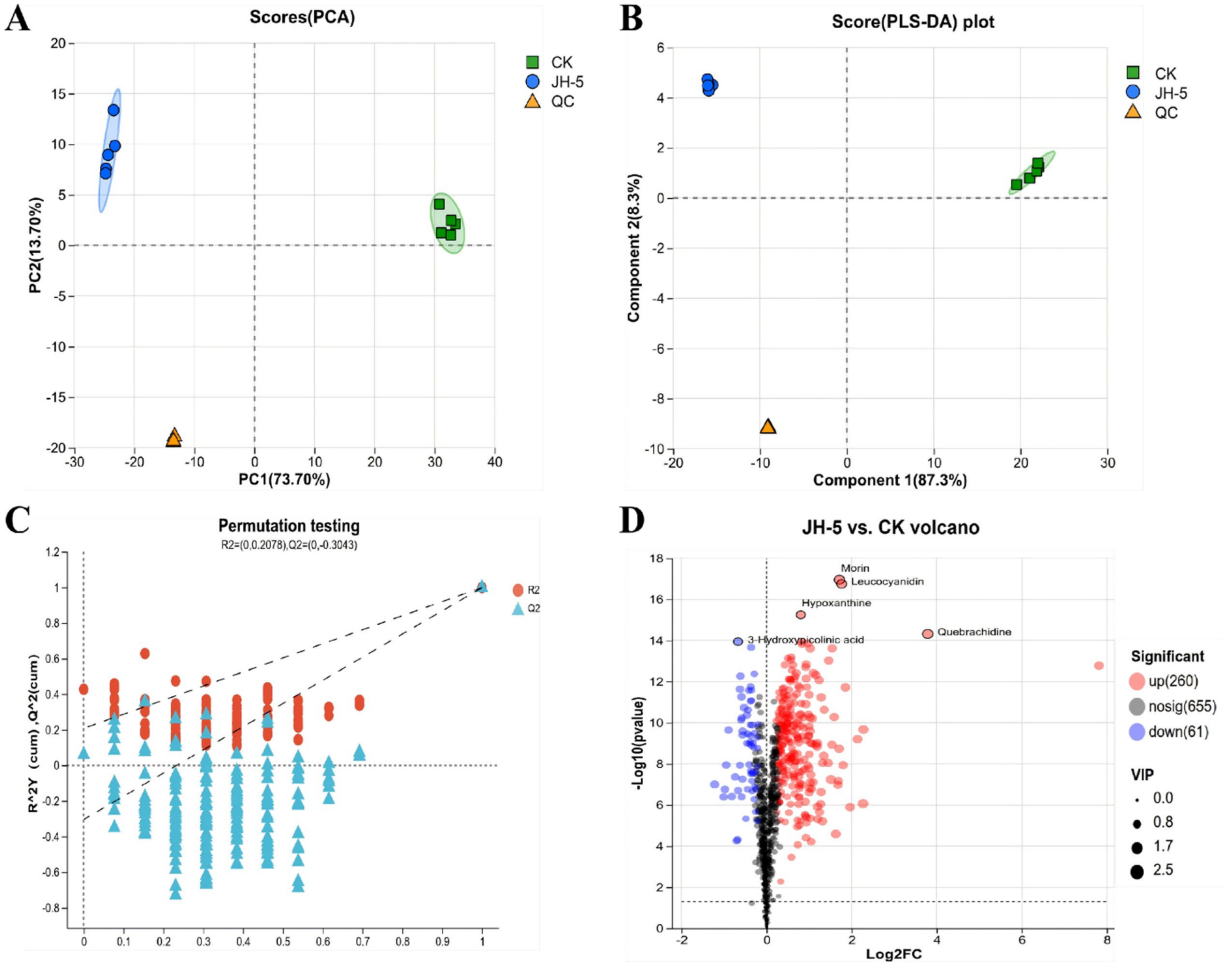
Metabolic changes overview of JH-5 ferment ginseng. **(A)** Principal component analysis (PCA) score plot showing the distribution of the control group (CK), JH-5 fermented group (JH-5), and quality control (QC) samples. **(B)** Partial least squares discriminant analysis (PLS-DA) score plot illustrating the distinct separation between CK and JH-5 groups. **(C)** Permutation test plot (*n* = 200) validating the robustness of the PLS-DA model. **(D)** Volcano plot of differential metabolites; red dots indicate significantly upregulated metabolites, and blue dots indicate downregulated metabolites in the JH-5 group compared to the CK group (VIP > 1, *p* < 0.05).

To explore the impact of JH-5 fermentation on the metabolic profile of ginseng, multivariate statistical analyses were performed on the metabolomics data. The Principal Component Analysis (PCA) score plot (Figure 6A) demonstrated a clear separation between the fermented (JH-5) and unfermented (CK) groups, with the first two principal components explaining 87.4% of the total variance (PC1: 73.70%, PC2: 13.70%). The tight clustering of Quality Control (QC) samples confirmed the stability of the instrument and the reliability of the data. Subsequently, a supervised Partial Least Squares Discriminant Analysis (PLS-DA) was applied to further distinguish the metabolic differences. As shown in Figure 6B, the two groups were distinctively separated along Component 1 (87.3%). The validity of the PLS-DA model was verified by a permutation test (Figure 6C), where the *Q*^2^ intercept was −0.3043, indicating the model was robust without overfitting. Finally, a volcano plot (Figure 6D) was generated to visualize the differential metabolites (VIP > 1, *p* < 0.05). Fermentation induced significant metabolic shifts, characterized by the upregulation of 260 metabolites and the downregulation of 61 metabolites. Key compounds, including Morin (VIP = 2.32, FC = 3.27, *p* < 0.001), Leucocyanidin (VIP = 2.18, FC = 3.40, *p* < 0.001), and Hypoxanthine (VIP = 1.8, FC = 1.75, *p* < 0.001), were found to be significantly enriched in the JH-5 group, highlighting the potential of this strain to enhance the bioactive composition of ginseng. The specific changes and their parameters can be found in the Supplementary Table 1.

#### 3.3.1 Altered the ginsenosides profile by JH-5

**Figure 7:**
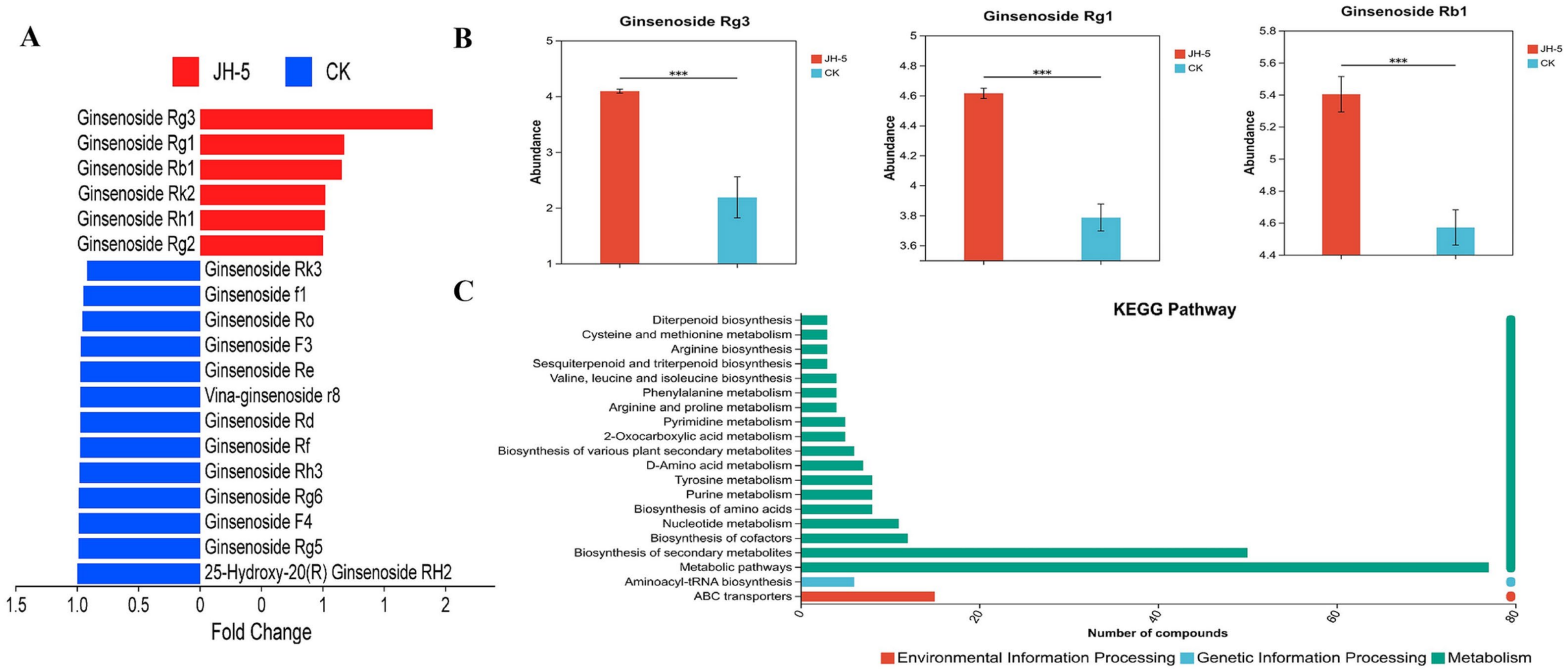
Profiling of ginsenoside variations in ginseng fermented by JH-5. **(A)** Bar chart illustrating the fold change (FC) of identified ginsenosides. Red bars represent upregulated ginsenosides (FC > 1), and blue bars represent downregulated ginsenosides (FC < 1) in the JH-5 group compared to the CK group. **(B)** Relative abundance comparison of key upregulated ginsenosides: Ginsenoside Rg3, Rg1, and Rb1. Error bars are presented as mean ± standard deviation. Statistical significance is indicated by asterisks (*** *p* < 0.001). **(C)** KEGG pathway enrichment analysis of differential metabolites before and after solid-state fermentation of *Panax ginseng* by Aspergillus cristatus JH-5. The bar colors represent the three main ontological categories: red for environmental information processing, blue for genetic information processing, and green for metabolism.

As ginsenosides are the primary pharmacologically active components of ginseng, their specific variations during fermentation were further investigated. As shown in Figure 7, the fermentation process by JH-5 induced a significant restructuring of the ginsenoside profile. Most notably, the fermentation significantly promoted the accumulation of rare ginsenosides. Ginsenoside Rg3, a highly bioactive rare saponin, exhibited the most substantial increase, while untargeted mass spectrometry is highly sensitive and detected trace background signals in the CK group (allowing the software to calculate a significant Fold Change of 1.87, *p* < 0.001), subsequent absolute quantification via HPLC confirmed that Rg3 was effectively absent (below LOQ) in the unfermented material but accumulated massively after fermentation. Statistical analysis confirmed that the abundance of Rg3 in the JH-5 group was significantly higher than in the CK group (*p* < 0.001). In addition to Rg3, the levels of major ginsenosides, specifically Ginsenoside Rg1 and Ginsenoside Rb1, were also elevated, showing fold changes of 1.26 and 1.17, respectively. Furthermore, minor increases were observed in other spooning, including Rk2, Rh1, and Rg2. In contrast, certain ginsenosides (such as Rk3, F1, and Ro) showed a decreasing trend (blue bars), which suggests a substrate-product relationship where specific ginsenosides are consumed or converted during the microbial metabolic process (Quan et al., 2013). Furthermore, our KEGG pathway analysis revealed significant enrichment in pathways directly related to ginsenoside metabolism, including Biosynthesis of secondary metabolites, Biosynthesis of various plant secondary metabolites, and Sesquiterpenoid and triterpenoid biosynthesis. Collectively, these results highlight the potent capability of the JH-5 strain to facilitate the biotransformation of ginseng saponins, particularly significantly enhancing the yield of high-value rare ginsenosides like Rg3, thereby potentially improving the therapeutic value of the fermented product.

### 3.4 Quantitative analysis of ginsenosides and protein

**Figure 8:**
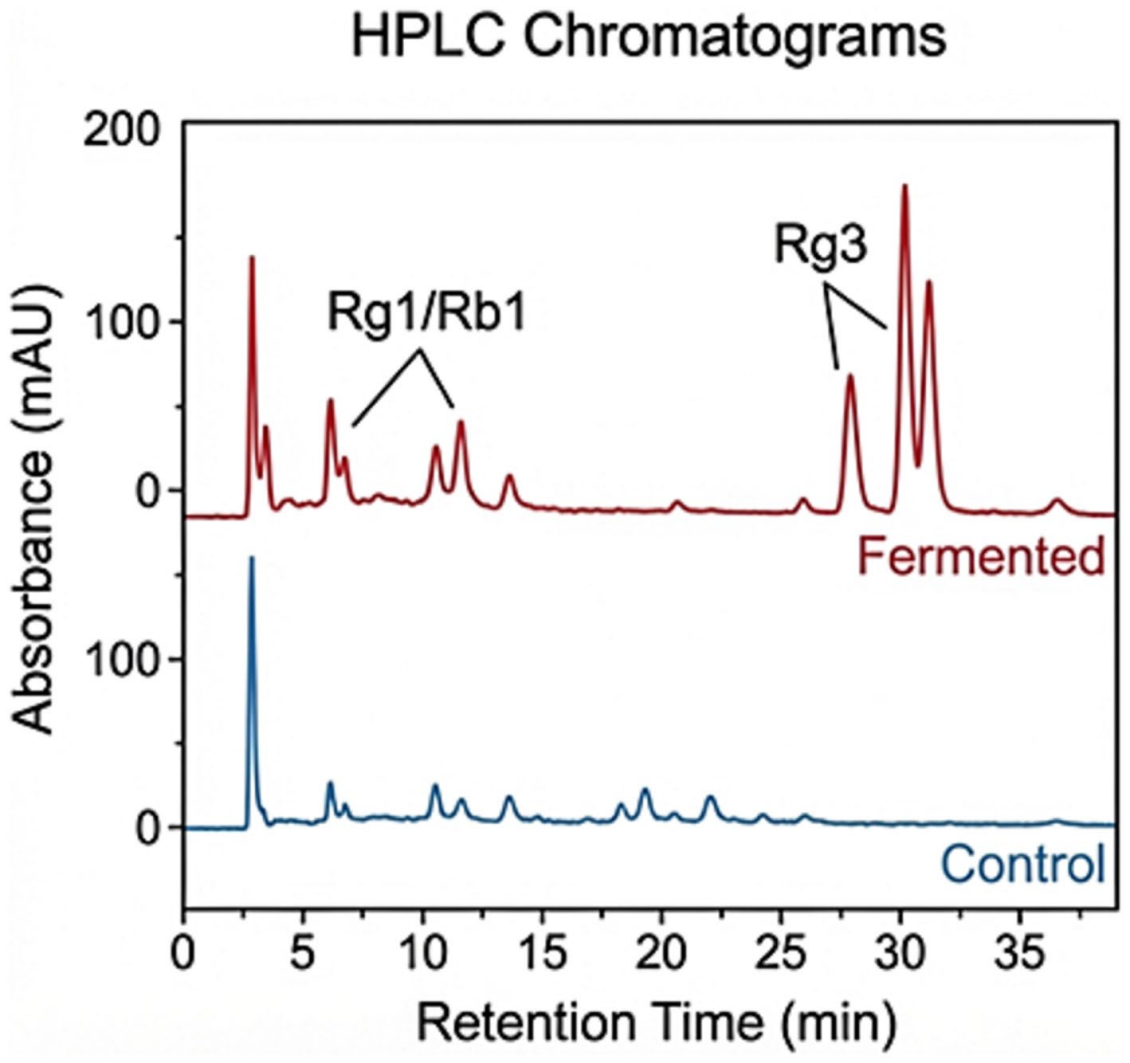
Comparative HPLC chromatograms and quantitative analysis of ginsenosides before.

To quantify the biochemical changes induced by fermentation, the contents of major and rare ginsenosides, along with total protein, were determined in the unfermented control (CK) and *A. cristatus* fermented ginseng (FG) using HPLC and the Kjeldahl method, respectively. As shown in Table 6, the fermentation process led to a significant 43.5% decrease in protein content, from 0.69% in CK to 0.39% in FG, indicating active hydrolysis of ginseng proteins by the fungus. Concurrently, the ginsenoside profile underwent a complex and beneficial transformation. Interestingly, rather than being depleted, the concentrations of the major ginsenosides Rg1 and Rb1 increased, with Rb1 showing a remarkable 280.6% rise. This phenomenon is likely due to a “concentration effect” caused by the fungal consumption of other substrates like carbohydrates, coupled with enhanced extraction efficiency from cell wall degradation. However, the most profound impact of the fermentation was the *de novo* generation of rare ginsenosides. As detailed in Table 7, the rare ginsenosides Rg3(S) and Rg3(R), which were not detected in the control group, accumulated to a total concentration of 0.23% in the fermented product. This accumulation of deglycosylated metabolites provides strong chemical evidence supporting the genomic prediction that *A. cristatus* JH-5 expresses active β-glucosidases capable of converting protopanaxadiol saponins, such as Rb1, into the more bioactive Rg3 forms (see Figure 8).

**Table 6:** Content of major ginsenosides and protein in unfermented and fermented ginseng.

Group	Ginsenoside Rg1 (%)	Ginsenoside Rb1 (%)	Protein (%)
Control (CK)	0.31 ± 0.02	0.31 ± 0.02	0.69 ± 0.03
Fermented (FG)	0.34 ± 0.02	1.18 ± 0.05	0.39 ± 0.02
Change (%)	+10.9%	+280.6%	−43.5%

Data are presented as mean percentages (g/100 g, dry weight basis).

**Table 7:** Content of rare ginsenosides in unfermented and fermented ginseng.

Group	Rg3 (S) (%)	Rg3 (R) (%)	Total Rg3 (%)
Control (CK)	N.D.	N.D.	N.D.
Fermented (FG)	0.01 ± 0.001	0.22 ± 0.01	0.23 ± 0.01

N.D., Not Detected, meaning the concentration was below the limit of quantification (LOQ <0.005%).

## 4 Discussion

This study successfully demonstrated that solid-state fermentation of *Panax ginseng* with *Aspergillus cristatus* JH-5 significantly enhances its bioactive profile, most notably through a substantial increase in rare ginsenosides. The integration of genomic, metabolomic, and quantitative HPLC analyses provides a comprehensive molecular basis for these observed changes, establishing a clear “gene-to-metabolite” narrative. Our findings not only validate the biotransformation potential of *A. cristatus* but also underscore its suitability as a robust and safe biocatalyst for producing high-value functional foods (Bai et al., 2025).

The remarkable accumulation of rare ginsenosides Rg3(S) and Rg3(R) is the cornerstone of this study and is strongly supported by the genomic blueprint of strain JH-5. The genome was found to be rich in genes encoding Carbohydrate-Active enZymes (CAZymes), particularly those from the Glycoside Hydrolase (GH) families. Within these, we identified numerous candidate genes for *β*-glucosidases (EC 3.2.1.21), which are widely recognized as the key enzymes responsible for the stepwise deglycosylation of major ginsenosides (Tran et al., 2023). Specifically, the conversion of the abundant ginsenoside Rb1 into the highly bioactive ginsenoside Rg3 requires the cleavage of the terminal glucose moiety at the C-20 position, a reaction catalyzed by specific β-glucosidases (Quan et al., 2012; Shin et al., 2015). The presence of these genes in the JH-5 genome provides a strong genomic basis for the observed biotransformation. However, it is important to acknowledge that the current “gene-to-metabolite” narrative is correlative. Future functional studies, such as *in vitro* enzymatic assays using heterologously expressed candidates (e.g., gene0147) or targeted gene knockout experiments, are required to definitively establish the causal role of these specific β-glucosidases in ginsenoside conversion. Furthermore, the identification of a diverse array of Cytochrome P450 genes suggests that *A. cristatus* JH-5 may also possess the capability to perform hydroxylations on the saponin aglycone, a mechanism known to diversify triterpenoid structures (Kim et al., 2015), though this warrants further investigation.

Beyond the generation of rare saponins, two other key phenomena were observed. First, the significant decrease in protein content aligns with the identification of numerous protease-encoding genes in the genome. This proteolytic activity likely breaks down ginseng proteins into smaller peptides and amino acids, which not only serve as a nitrogen source for the fungus but may also enhance the nutritional value and digestibility of the final product (Wang et al., 2016). Second, the counterintuitive increase in the concentrations of major ginsenosides Rg1 and Rb1 can be attributed to a “concentration effect.” As *A. cristatus* consumes primary metabolites like carbohydrates and starch for its growth, the total dry mass of the substrate decreases, thereby increasing the relative concentration of more stable secondary metabolites like ginsenosides (Ma et al., 2022). This “concentration effect” is a widely documented phenomenon in microbial solid-state fermentation, where the preferential microbial degradation of bulky primary matrices (e.g., cellulose, hemicellulose, and starches) naturally concentrates the remaining non-metabolized secondary compounds (Martins et al., 2011). This effect is likely amplified by the enzymatic degradation of ginseng’s complex cell wall structure by other CAZymes (e.g., cellulases and pectinases), which improves the extraction efficiency of these compounds during analysis (Srivastava et al., 2019).

A critical aspect of utilizing microorganisms for food applications is their safety profile. *A. cristatus*, as the dominant fungus in Fuzhuan brick tea, has a long history of safe consumption, granting it a status analogous to “Generally Recognized as Safe” (GRAS) (Rui et al., 2019). Our genomic analysis strongly corroborates this safety profile. A thorough search revealed no gene clusters associated with the biosynthesis of major mycotoxins such as aflatoxins, ochratoxins, or fumonisins. This finding is consistent with previous genomic studies on other *A. cristatus* strains, which also reported an absence of these carcinogenic biosynthetic pathways (Ge et al., 2016; Guo et al., 2022). Furthermore, extensive toxicological studies have repeatedly confirmed the safety of *A. cristatus*. It exhibits no acute or subacute toxicity in animal models, reinforcing its historical “Generally Recognized as Safe” (GRAS) status in food processing (Chan et al., 2019). While the absence of these biosynthetic gene clusters in antiSMASH predictions strongly supports the strain’s safety, genomic prediction has its limitations and cannot rule out the expression of unknown toxins. Therefore, to fully guarantee product safety, future studies must incorporate direct targeted LC–MS/MS screening for common Aspergillus-associated mycotoxins in the final fermented ginseng products. Nevertheless, the combined genomic data and its historical usage status position strain JH-5 as a highly promising biocatalyst with a favorable preliminary safety profile for industrial applications.

In conclusion, this study provides robust, multi-omics evidence that *A. cristatus* JH-5 is an effective and safe agent for the biotransformation of ginseng. By linking its rich genomic arsenal of hydrolytic enzymes directly to the targeted enrichment of rare ginsenosides Rg3, we have elucidated the molecular mechanism underlying its value-adding potential. However, this study is primarily correlational. Future research should aim to confirm these findings through functional genomics, such as gene knockout or heterologous expression of the identified β-glucosidase candidates, to definitively establish their roles (Cui et al., 2013; Du et al., 2014). Furthermore, optimizing the solid-state fermentation parameters (e.g., moisture, temperature, and fermentation time) could further enhance the yield of specific high-value compounds like ginsenoside Rg5 (Kim et al., 2000). However, translating this laboratory-scale success into industrial production requires addressing the inherent challenges of scaling up solid-state fermentation. Key engineering hurdles include maintaining substrate homogeneity, controlling metabolic heat generation, and ensuring uniform oxygen transfer across large bioreactor beds. Future bioprocess engineering studies focusing on dynamic moisture control and forced-aeration bioreactor designs will be crucial to guarantee batch-to-batch consistency and maximize bioconversion efficiency at a commercial scale. Ultimately, this work lays a solid theoretical foundation for the industrial development of novel, high-potency fermented ginseng products with improved therapeutic properties.

## Data Availability

The datasets presented in this study can be found in online repositories. The names of the repository/repositories and accession number(s) can be found in the article/supplementary material.

## Footnotes

1
http://soap.genomics.org.cn
